# Tactile Robotic Skin with Pressure Direction Detection

**DOI:** 10.3390/s19214697

**Published:** 2019-10-29

**Authors:** Jan Klimaszewski, Daniel Janczak, Paweł Piorun

**Affiliations:** 1 Institute of Automatic Control and Robotics, Faculty of Mechatronics, Warsaw University of Technology, A. Boboli 8 St., 02-525 Warsaw, Poland; 2 Institute of Metrology and Bioengineering, Faculty of Mechatronics, Warsaw University of Technology, A. Boboli 8 St., 02-525 Warsaw, Poland; d.janczak@mchtr.pw.edu.pl; 3 Student at the Faculty of Mechatronics, Warsaw University of Technology, A. Boboli 8 St., 02-525 Warsaw, Poland; pawelpiorun@interia.pl

**Keywords:** pressure sensor, graphene nanoplatelets, robotic skin, tactile sensor, sheer force detection

## Abstract

Tactile sensing is the current challenge in robotics and object manipulation by machines. The robot’s agile interaction with the environment requires pressure sensors to detect not only location and value, but also touch direction. The paper presents a new, two-layer construction of artificial robotic skin, which allows measuring the location, value, and direction of pressure from external force. The main advantages of the proposed solution are its low cost of implementation based on two FSR (Force Sensitive Resistor) matrices and real-time operation thanks to direction detection using fast matching algorithms. The main contribution is the idea of detecting the pressure direction by determining the shift between the pressure maps of the skin’s upper and lower layers. The pressure map of each layer is treated as an image and registered using a phase correlation (POC–Phase Only Correlation) method. The use of the developed device can be very wide. For example, in the field of cooperative robots, it can lead to the improvement of human machine interfaces and increased security of human–machine cooperation. The proposed construction can help meet the increasing requirements for robots in cooperation with humans, but also enable agile manipulation of objects from their surroundings.

## 1. Introduction

Robotic manipulators have been widely used in industry, medicine, entertainment, etc. for over 50 years. In recent years a new trend has emerged—cooperative robotics. It aims to facilitate human–machine cooperation and introduce robots to the direct human space while maintaining safety rules. To achieve these goals, it is necessary to develop more natural human–machine interfaces. In addition to ensuring human safety, these interfaces will allow the machine to be equipped with more agile functions and skills. Much development research is being conducted in the field of robotic skin, which is to implement a natural touch interface. One aspect of this interface is the detection of human touch by a robot. Another aspect is for the robot to know how much force it exerts on the environment. The robotic skin described in this article allows to equip the robot with the ability to detect touch and it is a good sensor for cooperative robots.

This article presents a new design of robotic skin enabling detection of not only the value and location of touch, but also its direction. This goal is achieved by using two layers, each of which measures the location and scalar value of the contact pressure using a matrix of resistance sensors. A new approach to touch direction estimation is accomplished by matching pressure maps from top and bottom layer of robotic skin. If external force is applied to the top layer at direction not normal to skins’ surface, the top layer will be displaced relative to the bottom layer. This means that similar pressure will be registered in different locations of the upper and lower sensor matrix. The pressure map of each layer is treated as an digital image, and the pressure direction is calculated by determining the shift between these images. The main advantages of the proposed solution are its low cost of implementation based on two simple FSR (Force Sensitive Resistor) matrices and real-time operation thanks to direction detection using fast matching algorithms.

As part of the research, a prototype robotic skin was designed and manufactured. The implementation of appropriate algorithms for processing data from two robotic skin layers was performed and the entire system was tested. The measurement results confirm the effectiveness of the developed device and methods.

The work layout is as follows. In Section 2, an overview of articles about robotic skin is presented. In Section 3, a new robotic skin device is presented and the measuring system is briefly described. In Section 4, the developed algorithm for estimating the location, value and pressure direction is presented. In Section 5, the measurement results of the developed prototype are presented. Finally, in Section 6, the achieved results are discussed and summarized.

## 2. Related Research

Many researchers have presented different approaches to solve the problem of tactile measurements in robotics. There are a large number of publications describing devices for measuring normal value and pressure location from the force exerted on the device, i.e., [1,2,3,4,5]. Fewer authors attempt to solve the problem of detecting the direction of pressure, i.e., [6,7,8,9,10].

A general overview of the application and classification of methods of functioning of robotic skin can be found, among others in [11].

In most cases, only the scalar pressure measurement and its location are performed without detecting the pressure direction. This is often done as: resistance measurement, capacity measurement [1,2], air pressure measurement [3] using the Hall effect, [4] or methods combining the advantages of different approaches [5]. Particular attention should be paid to [5]—a sensor has been described there with a design taking into account, as in our work, the use of a resistance matrix based on graphene. The skin described in the article is a combination of a measurement system based on resistance and capacity. The capacitive sensor uses a mixture of silicone rubber and “carbon black” (CBSR). The article contains basic tests of the developed device without demonstrating a specific application.

In [12], a solution to the problem of measuring pressure force and pressure surface with the use of a two-layer skin based on capacity measurement is presented.

In [13], the advantages of resistance sensors were emphasized: a simple design and a simple measurement method based on a change in resistance under pressure, easy prototype construction even using flexible PCBs. From the electronics side, the measurement method uses only a voltage divider and an analog-to-digital converter. These are low-cost sensors that provide low-noise measurements with good sensitivity. The disadvantages are the presence of hysteresis in operation, relatively high power consumption and limited durability of the sensors (material). In [14], ways of using these types of sensors for the implementation of soft grippers were described. Many publications deal, in particular, with the implementation of touch for a robotic hand [1,3,4]. The authors of [15] present possible applications for soft robotics in biomedical applications. A broad review of the methods of construction and application of robotic skin can also be found in the PhD dissertation [16].

These sensors, however, are also used outside the field of soft robotics. Many authors try to use robotic skin to calibrate the kinematic chain for rigid mechanisms (especially for the iCub robot (http://www.icub.org/)) [17,18]. An interesting solution is to supplement the calibration process with data from the stereo camera as described in [17]. Other authors use tactile sensors, e.g., for 3D reconstruction of the surface touched by the manipulator [19,20]. In these publications, it is worth paying special attention to the modular structure of the skin and the use of gravity vector information for each module.

The construction of a single layer of the sensor matrix making up the skin and the idea of measurement similar to that described in this article can be found, in particular, in [21,22,23]. The authors of [21] present a physically robust and flexible construction, whereas the authors of [22,23] present ways to use a similar sensor to control an industrial robot. Other examples of industrial robot control are shown in [24,25], where multi-touch was used. The sensor shown in [19,20] was used there. An interesting application of the robotic skin is presented in [26], where a controller based on the neural network for controlling the mechanical impedance of the manipulator is described. The network inputs are pressure measurements from robotic skin, the output is the estimation of moments to the admittance regulator. The skin was made using a piezoresistive sensors matrix.

Other interesting articles about the robotic skin are [27,28], where flexible, stretchy skin is described and pressure location measurement is made using only five electrodes.

A smaller number of authors undertook to construct devices that perform a more difficult task—detecting not only the value and location, but also the direction of exterted force. Multi-axis pressure measurement is often intended for use on robotic hands [29,30].

Initially, the task of detecting the direction of exterted force was implemented using strain gauges [31,32,33]. This straightforward approach to the problem of multi-axis pressure measurement is to use strain gauge-based sensors integrated into the robot structure. However, such solutions are difficult to integrate into small sizes that allow robotic skin implementation with distributed force measurements [33]. For example, the hand MAC [31] and Robonaut 2 [32] have been equipped with strain gauges not only at the fingertips, but also in the earlier parts of hand. A similar solution—based on three-axis measurement using strain gauges—was also described in soft skin of the robot Macra [33]. This solution, however, is characterized by a relatively large size and is difficult to use, e.g., in fingertips.

Small sensors can be manufactured on the basis of MEMS technology, e.g., triaxial measurement in [34], and measurement in [35] using four triaxial sensors.

Another technology that enables multi-axis measurement is the use of piezoelectric elements. Touchence (http://www.touchence.jp/en/) produces 3-axis tactile sensor based on piezoelectric elements.

Robotic skin applications that require significant accuracy are based on optical measurements [36,37,38]. Unfortunately, they are also characterized by a considerable degree of complexity and a relatively large size. Multi-axis touch force sensors using cameras can also be integrated, e.g., into the fingertips of a robotic hand, but are usually too thick for use with robotic skin. A preliminary solution to the problem of optical sensor size is given in [39], but this idea was not developed in further publications. The 3-axis optical sensor described in [33] was integrated into the soft flesh robot and implemented into production by Touchence. OptoForce (http://optoforce.com/) sells a smaller (10 mm wide and 8 mm high) version of the sensor based on a similar principle of operation.

New publications in the field of robotic skin for multi-axis measurement usually focus on the use of magnetic sensors [6,7,8,40,41] or on the construction of skin with spatial bumps or other non-flat structure elements [9,10].

The authors of [41] describe a matrix of 16 triaxial pressure sensors for robotic hands. Each of them is similar to a single sensor described in [4]—it measures exerted force using the Hall effect with the MLX90393 electronic chip. In [41], each sensor has I2C digital interface, and each sensor module is connected to four I2C buses, requiring only seven wires for each module. The triaxial taxels are quite small and close to each other (4.7 mm from the center of one taxel to the next). Other publications, showing the possibilities of using magnetic measurements in the construction of pressure sensors, are presented, i.e., in [42,43,44].

A similar construction to [41] is presented in [6], where the sensor matrix on the robotic hand fingertip is described. Each sensor works on the same principle as in [41]. In [7], the same sensors were used for the iCub robot.

Multi-axis pressure measurement was carried out similarly in [8]. Publication describes a single 3-axis sensor based on the Hall effect using the MLX90393 chip. Very similar solutions are presented in [40,45].

The authors of [46] described hybrid measurement module consisting of a matrix of 32 pressure sensors and one 9-DoF MARG sensor (magnetic, angular rate, and gravity). Place the sensors within compliant structure, which is similar to our solution.

Less complicated solutions are based on the spatial configuration of the skin in the form of various types of bumps. In [47,48], a bump was added on top of an array of four capacitive sensors to make the array sensitive to shear forces. In [9], the detection of the direction of touch, whether the skin is stretched or bent, is presented. This was accomplished on the basis of two layers equipped with tabs from above and below. The idea of measurement is similar to that in [10]. The authors of [10] describe a matrix of capacitors for measuring normal and skewed forces. The structure of robotic skin is spatial (tumors and bumps) and a matrix of flat sensors is spread on it. The device application described is the fingers of a robotic hand. W [49] also uses a spatial structure with a single piezoelectric sensor. Based on the measurements, normal force, shear force, and bending, along with temperature are determined.

Another interesting approach to the problem of robotic skin with multi-axis pressure measurement is capacitive skin sensors. They can measure the shear forces without a bump or plate as described in [50,51]. Unfortunately, described device does not include the measurement electronics. Therefore, its use for distributed sensing in robotic skin is not straightforward. Another solution based on capacitive measurements is described in [52]—a capacitive 3-axis sensor embedded in soft silicone.

## 3. Hardware Design

### 3.1. New Double-Layer Robotic Skin

**Figure 1:**
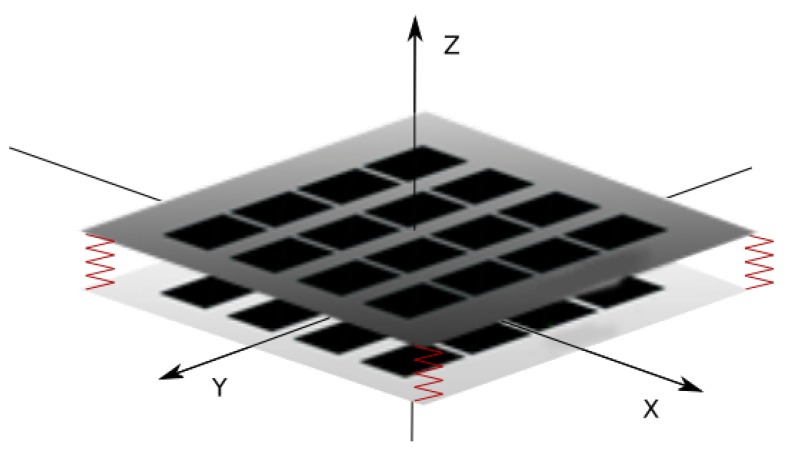
Diagram of the double-layer FSR (Force Sensitive Resistor) matrix.

The new skin structure described in this article consists of two layers of force-sensitive resistors (FSR) matrices separated from each other by a elastic element. The structure of the two-layer skin is schematically illustrated in Figure 1. Each layer is made of a matrix of individual sensors and allows to register a pressure map from external forces. The direction of force is detected based on the calculation of translational shift between maps from the upper and lower layers. As part of the work on this article, a prototype device was made with 16 × 16 sensors in each layer. The size of a single sensor is approximately 5 × 5 mm. To the best of the authors’ knowledge, no other implementation of robotic skin based on a similar principle was found.

**Figure 2:**
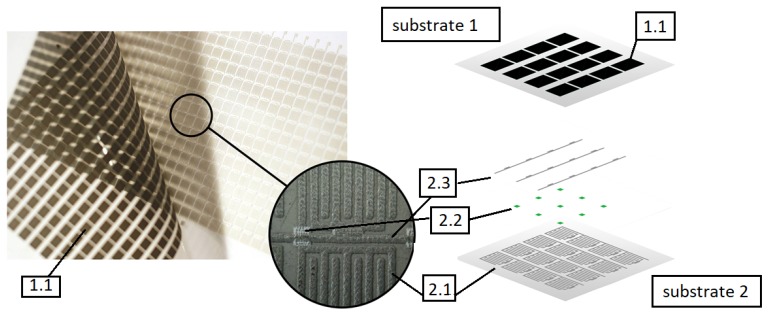
Single robotic skins’ layer—the FSR matrix real view (left) and diagram (right); 1.1—resistive layer on substrate 1; on substrate 2: 2.1—comb electrodes; 2.2—dielectric separators; 2.3—paths connecting sensors in rows.

Each layer is constructed of FSR sensors connected in a rectangular matrix. Each resistance sensor is based on graphene nanoplatelets with comb electrodes and is similar as described in [53]. Using graphene nanoplatelets has a few main advantages. As demonstrated by previous research, resistive pressure sensors containing in the sensory layer carbon fillers such as graphene nanoplatelets or carbon nanotubes show very little hysteresis [54,55]. Multiple dynamic tests involving cyclical load of the sensor did not show any sensor characteristics drift. The single layer of FSR-resistive sensors shown in Figure 2 was screen printed on two substrates as shown in [53]. The robotic skin single layer consists of a condictive layer of comb electrodes (labeled 2.1) printed on plastic foil (substrate 2), connected along columns, dielectric separators (2.2), path layers connecting sensors in rows (2.3), and resistive layer (1.1) printed on another plastic foil (substrate 1). The prototype of a single layer of robotic skin is presented on the left of Figure 2. Each of the columns and rows is powered separately using dedicated electronic driver, making it possible to connect the measuring signal to a single sensor.

**Figure 3:**
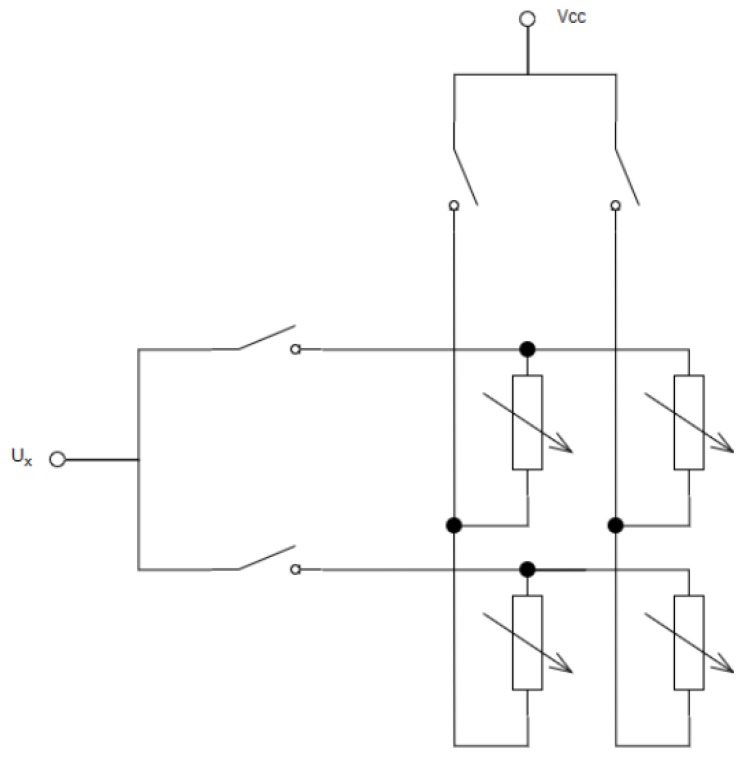
Measurement principle for FSR matrix of size 2 × 2.

A single FSR layer combines individual FSR sensors into columns and rows. So as to supply the signal to only one sensor, the circuit consisting of only one column and one row is closed. An example of the 2 × 2 matrix structure is shown in Figure 3.

**Figure 4:**
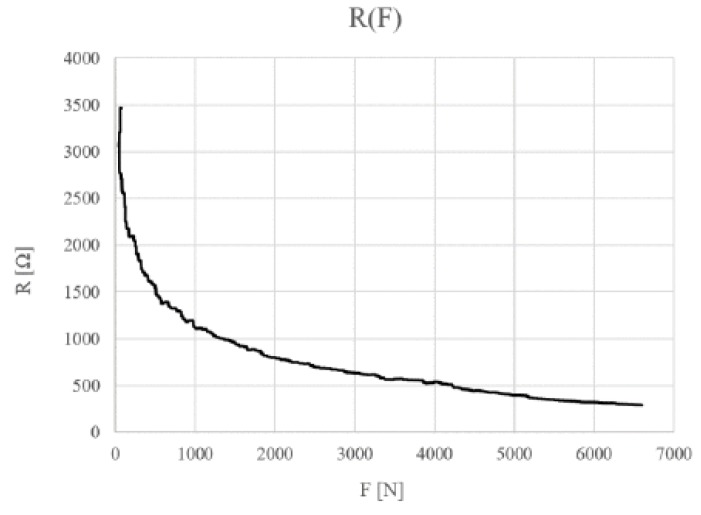
Characteristics of the FSR sensor resistance to pressure (2%wg GNP/PVDF).

Larger layers are constructed in a similar way. The resistance of FSR sensors depends on the material composition of which the sensor electrodes and conductive layer are made. Sensors with 300–3500 ohm resistance are used in this article. The force to resistance F(R) characteristic of this sensor is shown in the graph below (Figure 4).

### 3.2. Measurement Circuit

**Figure 5:**
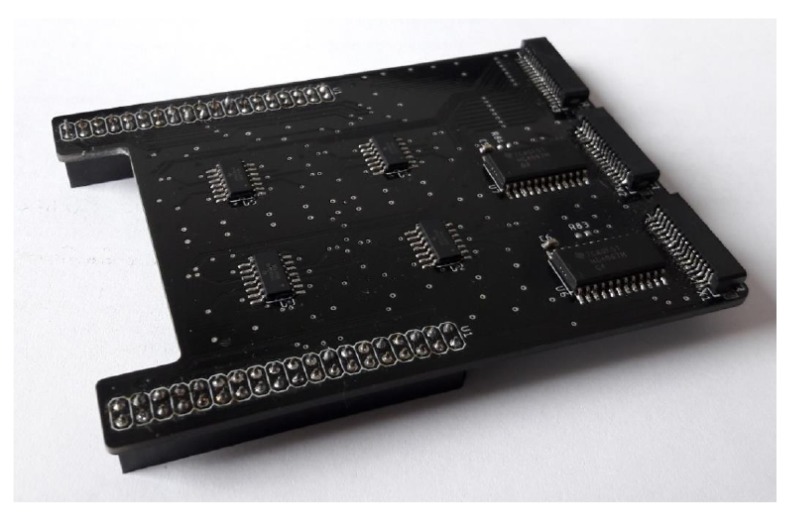
Expander board after assembly—top view.

As part of previous work, a electronic measuring board was developed as an expander of the Nucleo-F446RE board. The measuring board is shown in the illustration Figure 5. Expanded Nucleo-F446RE board measures the pressure value for each sensor of two layers of the new robotic skin structure, and then sends them to a PC. The communication is carried out with USART over the serial port. To estimate the pressure direction, further analysis of the transmitted data is performed on a PC.

To measure the pressure force on the FSR sensor, which is part of the new design, the measurement of the sensor voltage in the voltage divider system was used. For each sensor, the measurement of the variable resistance of the $R_{f}$ sensor is done indirectly by measuring the voltage at a series connected resistor $R_{x}$ with a known resistance.

The voltage on the sensor was adapted to the input voltage levels of the microcontroller ADC transducers using a set of 16 differential amplifiers. The block of differential amplifiers, which receive signals from FSR matrix lines and the reference voltage, consists of four 4-channel OPA4354 operational amplifiers.

The FSR matrix is attached through 1 mm FFC tapes to the connectors on the expander board.

The developed device acquires the pressure map from both measuring layers at a rate of ~30 frames per second, which is equivalent to one measuring cycle every 30 ms. Several components affect this speed. Among others, they are microcontroller clock speed, the speed of measuring the analog signal from the ADC converter, the speed of data transfer through the serial port, and the speed of data processing by the computer for visualization.

The rate of pressure map acquisition can be further reduced. Preliminary acquisition time studies have shown that the most time consuming process is to receive and convert measurement data into an image for visualization. The microcontroller clock frequency of 180 MHz enables ~225 million operations per second. The time of a single measurement of the signal from the ADC converter is from 1 to 16 μs. For 512 sensors in matrix it gives approx. 0.5 to 8 ms. Therefore, when configuring ADC transducers, one must strive to minimize measurement time while maintaining reliable results. With a good configuration of the microcontroller peripherals, it can be assumed that one series of measurements for sensor matrix by a microcontroller lasts no more than a few milliseconds (i.e., potentially over 100 series per second). The acceleration of such a system may be accomplished by the optimization of the program code on the PC.

The measuring signal from 16 channels is internally multiplied by a microcontroller equipped with 3 ADC converters. In the developed implementation, one ADC transducer is used at the same time, so only one channel was measured at a time. Acceleration of measurement data acquisition is possible by modifying the measurements in such a way as to use as many ADC transducers as possible to carry out measurements simultaneously. Further, in microcontrollers containing the direct memory access (DMA) module, measurements from ADC converters can be processed much faster. Despite the presence of this module in the STM32-F446RE microcontroller used, it was not included in the program.

## 4. Measurement Algorithm

**Figure 6:**
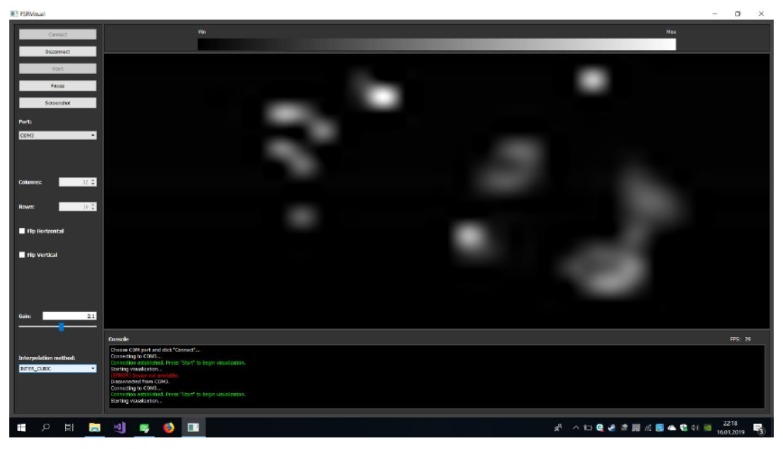
An example of visualization of measurement data from one layer (one FSR matrix).

As already mentioned, the electronic measuring system performs measurements of the scalar pressure value for each sensor of two layers of the new structure, and then sends them to a PC. To estimate the pressure direction, the computer analyses pressure measurements from electronic device. The application developed on a PC also implements visualization of measurement data from each layer. An example of visualization of measurement data from one layer is shown in Figure 6.

**Figure 7:**
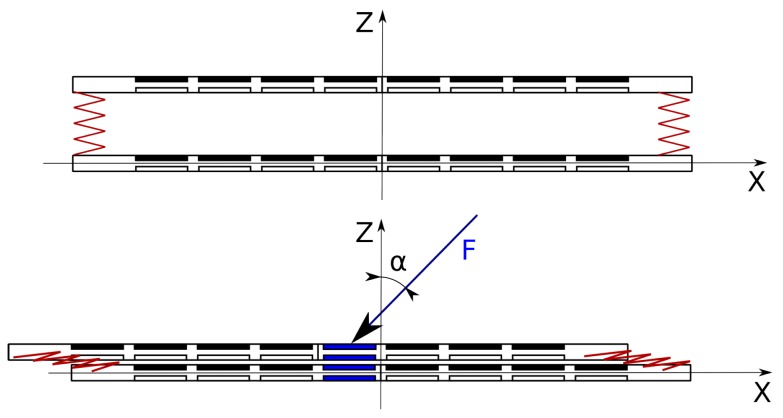
The idea of pressure vector estimation.

The idea of pressure direction estimation is presented in the Figure 7. If external force *F* is applied to the top layer at an angle α with respect to *Z* axis, the top layer will be displaced relative to the bottom layer. This means that similar pressure will be registered in different locations of the upper and lower sensor matrix. The direction of displacement depends on the angle β of projection of force *F* on the XY plane with respect to *X* axis. Measuring this displacement will allow estimating the direction of force in XY plane.

The measurement data from the lower layer $r_{b}$ and the upper layer $r_{t}$ are saved as matrices of scalar values in the Cartesian two-dimensional XY coordinate system associated with the layer. So as to quickly estimate the pressure direction, the phase correlation (POC–Phase Only Correlation) method [56,57] was used. The method is commonly used in the field of image processing to determine the offset between two similar [58] and sometimes multimodal [59,60] images. Image processing methods have already been used to determine the type of touch, e.g., in [38], but only for video data—camera-based touch. The use of phase correlation is new in the area of robotic skin pressure direction detection. This method has been used in the article to estimate the translational shift between two similar pressure maps. Below is a detailed presentation of the method in the discussed scope.

Let r(x,y) represent a map of scalar pressure values of MxN. The discrete Fourier transform of this map can be written as follows [61],

(1) $$\begin{array}{c} R\left(u,v\right)=F\left(r\left(x,y\right)\right)=\sum_{x=0}^{M-1}\sum_{y=0}^{N-1}g\left(x,y\right)e^{-j2\pi (\frac{ux}{M}+\frac{vy}{N})}=\\ \left|R\left(u,v\right)\right|e^{-j\Phi (u,v)} \end{array}$$

where,

x,y—coordinates of the location of the map pressure;
u,v—coordinates of Fourier transform coefficients;
|R(u,v)|—signal amplitude in the frequency domain—characterizes the frequency of each of the map pressure levels; and
Φ(u,v)—signal phase in the frequency domain—characterizes the spatial structure of the pressure map.

If we have two maps $r_{b}\left(x,y\right)$ and $r_{t}\left(x,y\right)$ that represent the same shape, but in different positions relative to the center of each map, you can write it as Equation (2):

(2) $$r_{b}\left(x,y\right)=r_{t}\left(x-x_{0},y-y_{0}\right)$$

where,

$\left(x_{0},y_{0}\right)$—unknown translational shift between pressure maps.

To determine the unknown translational shift $\left(x_{0},y_{0}\right)$ it is possible to use the phase correlation method based on Fourier transform. Using Equation (2), and according to the Fourier shift theorem, we can write

(3) $$R_{b}\left(u,v\right)=R_{t}\left(u,v\right)e^{-j2\pi (\frac{ux_{0}}{M}+\frac{vy_{0}}{N})}=G_{1}\left(u,v\right)e^{j\Delta (u,v)}$$

where,

$R_{b}\left(u,v\right)=F\left(r_{b}\left(x,y\right)\right)$
$R_{t}\left(u,v\right)=F\left(r_{t}\left(x,y\right)\right)$
$\Delta \left(u,v\right)=-2\pi \left(\frac{ux_{0}}{M}+\frac{vy_{0}}{N}\right)$

Referring further to the form Equations (1) and (3), we can additionally determine the following relationships,

(4) $$\begin{array}{c} R_{b}\left(u,v\right)=\left|R_{b}\left(u,v\right)\right|e^{-j\Phi_{b}\left(u,v\right)}\\ R_{t}\left(u,v\right)=R_{b}\left(u,v\right)e^{j\Delta (u,v)}=\\ |R_{b}\left(u,v\right)|e^{-j\Phi_{b}\left(u,v\right)}e^{j\Delta (u,v)}=\left|R_{b}\left(u,v\right)\right|e^{\left(-j\Phi_{b}\left(u,v\right)-\Delta \left(u,v\right)\right)} \end{array}$$

To emphasize the Δ component containing information about the translational shift $\left(x_{0},y_{0}\right)$, one can determine the normalized spectral power density of maps $r_{b}$ and $r_{t}$. It is defined as follows,

(5) $$P_{w}\left(u,v\right)=\frac{R_{b}\left(u,v\right)R_{t}^{*}\left(u,v\right)}{|R_{b}\left(u,v\right)R_{t}^{*}\left(u,v\right)|}$$

where, $R_{t}^{*}\left(u,v\right)$—complex conjugate of $R_{t}\left(u,v\right)$.

Using Equation (4), we can find Formula (5) as

(6) $$\begin{array}{c} P_{w}\left(u,v\right)=\frac{|R_{b}\left(u,v\right)|e^{-j\Phi_{b}\left(u,v\right)}\left|R_{b}\left(u,v\right)\right|e^{j(\Phi_{b}\left(u,v\right)-\Delta \left(u,v\right))}}{\left|\right|R_{b}\left(u,v\right)|e^{-j\Phi_{b}\left(u,v\right)}|R_{b}\left(u,v\right)\left|e^{j(\Phi_{b}\left(u,v\right)-\Delta \left(u,v\right))}\right|}\\ =\frac{|R_{b}{\left(u,v\right)|}^{2}e^{-j\Phi_{b}\left(u,v\right)}e^{j(\Phi_{b}\left(u,v\right)-\Delta \left(u,v\right))}}{|R_{b}{\left(u,v\right)|}^{2}}\\ =\frac{|R_{b}{\left(u,v\right)|}^{2}e^{-j\Delta (u,v)}}{|R_{b}{\left(u,v\right)|}^{2}}\\ =e^{-j\Delta (u,v)} \end{array}$$

Then, we can calculate the inverse Fourier transform of $P_{w}$:

(7) $$p_{w}\left(x,y\right)=F^{-1}\left(P_{w}\left(u,v\right)\right)=F^{-1}\left(e^{-j\Delta (u,v)}\right)=\delta \left(x-x_{0},y-y_{0}\right)$$

where, $\delta (x-x_{0},y-y_{0})$—Dirac delta with location $\left(x_{0},y_{0}\right)$.

Using the results of the Equation (7), it is possible to determine the shift between the pressure maps $r_{b}$ and $r_{t}$ by finding the location of the maximum value in $p_{w}$ from Formula (7).

For the purpose of the implementation method described above, the OpenCV (https://opencv.org/) library was used, which contains the implementation of the phase correlation method. Based on the translational shift, one can estimate the direction in the XY plane of external force *F*. This direction is uniquely determined by the *v* vector according to the relationship

(8) $$v=(x_{0}-x_{p},y_{0}-y_{p})$$

where, $\left(x_{p},y_{p}\right)$—calibration coordinates of the robotic skin, characterizing the shift between the upper and the lower sensor layer under external force *F* parallel to the axis *Z* ($\alpha =0^{\circ }$).

The direction in the XY plane of the external force *F* can easily be determined from *v* using standard trigonometric functions. Suppose this direction can be characterized by the angle β measured between the *v* vector and the *X* axis in the XY plane.

The results of this method are presented in Section 5.

## 5. Test Results

### 5.1. Hardware Set-Up

**Figure 8:**
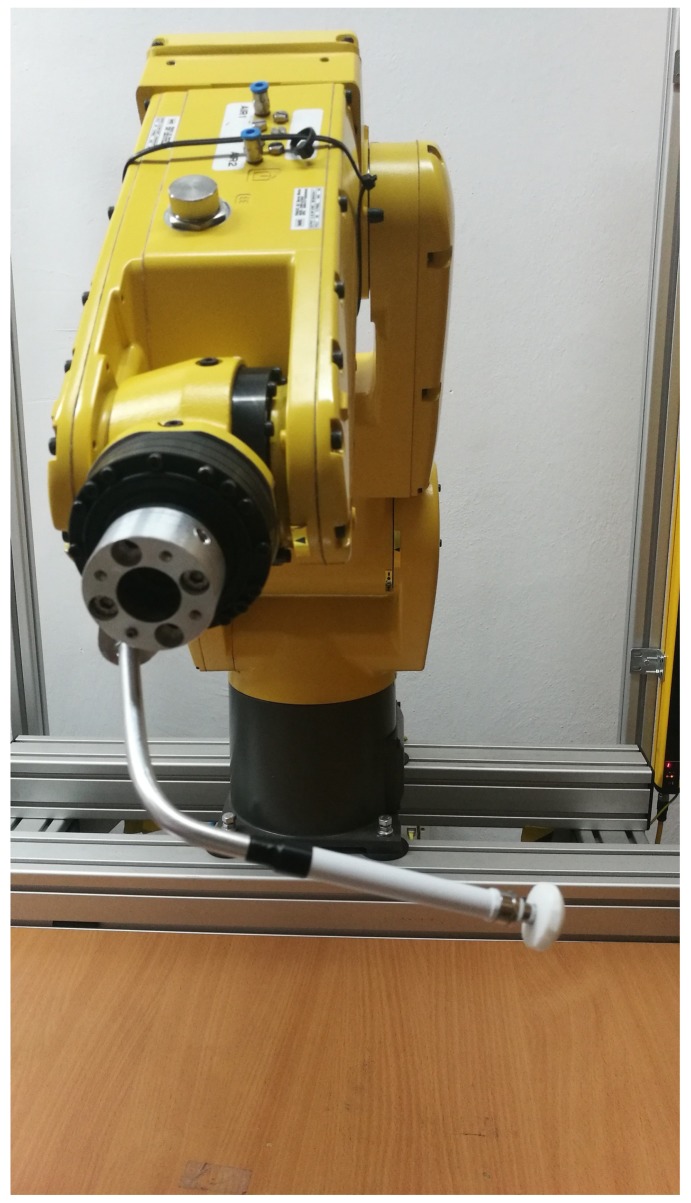
Robot with pressing tool.

As part of the tests, an experiment was carried out using the Fanuc industrial robot. A robotic tool was designed for the robot to simulate single point pressure. The tip of the tool was made as a flexible silicone element. The experiment consisted of repetitive, performed by the programmed robot, applying external force with a varying angles, α and β. The robot with the tool is shown in the illustration Figure 8.

The tests carried out consisted of external force vector varying in each of nine directions: parallel in the *Z* axis ($\alpha =0^{\circ }$) and at an angle of $45^{\circ }$ to the *Z* axis ($\alpha =45^{\circ }$) in eight XY plane directions distributed symmetrically every $45^{\circ }$ ($\beta =0^{\circ },45^{\circ },90^{\circ },135^{\circ },180^{\circ },225^{\circ },270^{\circ },315^{\circ }$). The α angle was determined using a simple protractor with $1^{\circ }$ intervals. Therefore, the accuracy of determining the alpha angle can be taken as $\pm 1^{\circ }$. The obtained results are presented in the next section.

### 5.2. Example Results

**Figure 9:**
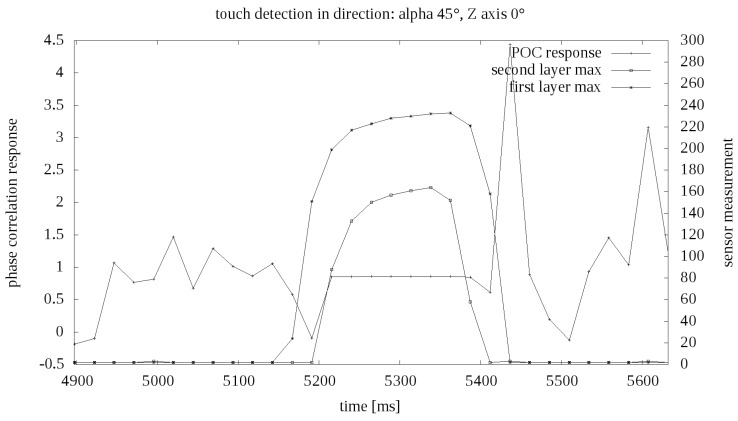
Example touch measurement in time.

Examples of pressure results in the function of time are presented in the graph Figure 9. On the value axis there are numerical indications from the analog to digital converter. No calibration was performed for the robotic skin prototype, so no specific values of external force are known. Based on general tests, it was established that the maximum value of force exerted on the matrix by the robot equals several dozen of Newtons.

**Figure 10:**
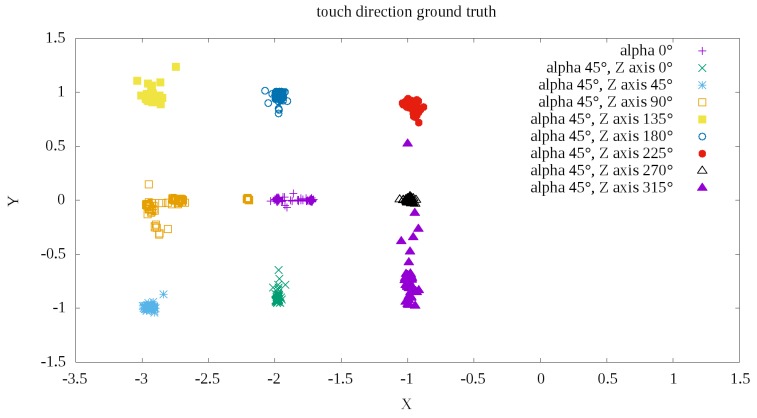
Example POC (Phase Only Correlation) measurements depending of touch direction.

The results of the algorithm described in the Section 4 are shown in the illustration of Figure 10. The colors indicate ground truth data on the direction of external force exerted by the Fanuc robot tool. At first glance, good segmentation and the ability to easily classify the direction of force based on measurements are apparent. Only the $\alpha =45^{\circ },\beta =315^{\circ }$ direction shows less precision than other measurements.

Finally, the mean measurement errors were determined as the angular distance of the vector determined according to Formula (8) relative to theoretically determined direction according to ground truth from the Figure 10. The error was determined in degrees as the difference in the angle of inclination between the line representing the ground truth direction and the line determined by the direction of measurement relative to the center of measurement. The center was defined as the mean measuring point $\left(x_{0},y_{0}\right)$ for the external force *F* exerted with $\alpha =0^{\circ }$. The mean errors and standard deviations are shown in Table 1.

### 5.3. Discussion

Based on the measurements, it can be determined that, using the prototype robotic skin, it is possible to distinguish one of the eight directions in the XY plane of exerted force *F* with the average accuracy and the average standard error of $3.72\pm 4.73{[}^{\circ }]$.

These results were certainly influenced by α angle accuracy. It was specified by the way the robotic skin was positioned relative to the industrial robot using a simple protractor. The main idea of described experiments was not to ensure high measurement accuracy, but to test the validity of the new idea of pressure direction measurement. The measurements carried out for each of the nine α directions are highly repeatable due to the use of a high class Fanuc industrial robot in the research.

It is a well known fact that resistance based pressure sensors have often a problem with a measurement drift effect. This effect occurs mainly at static loads. As a result of the procedure used to conduct the experiment, the tested robotic skin was subjected only to dynamic loads. In [54,55], it was shown that the use of graphene nanoplatelets as a resistance pressure sensor in such conditions shows very little hysteresis and a stable pressure characteristics.

## 6. Conclusions

The article describes the developed structure of robotic skin for measuring location, value of pressure and its direction with the average accuracy and the average standard error of $3.72\pm 4.73{[}^{\circ }]$. The presented measurement idea was confirmed based on the measurement results obtained from the prototype design of the device.

More precise measurement based on this technology is possible, but requires higher resolution of each upper and lower layer of robotic skin. Higher resolution will also reduce the thickness of the skin and possibly allow measuring of the angle, α, of the force, *F*, exerted on the surface of the skin.

As the next stage of research, calibration of robotic skin measurements is planned. It will allow determination of the measurement characteristics of the device (exerted pressure in function of resistance). Despite the preliminary discussion in [54,55] an additional direction of research in this area may be checking the device’s measurement drift under static load conditions. Future research should also include the development of a better method for calibrating the position of the robotic skin relative to the industrial robot coordinate frame. It should also increase pressure direction measurements accuracy.

As part of further work, we plan to increase the resolution and reduce the size of a single robotic skin cell. This will lead to device thickness reduction. When increasing the robotic skins’ layers resolution, the problem of processing algorithms execution time have to be addressed—both the number of individual sensors to collect measurements from and the size of the pressure maps to be registered using phase correlation will increase. It will be necessary to optimize the code responsible for measuring and processing measurement data, as well as to modify the measuring device.

## Figures and Tables

**Table 1:** Direction detection errors summary.

Ground Truth $\beta \;[{}^{\circ }]$	Mean Error $\left[{}^{\circ }\right]$	Standard Deviation $\left[{}^{\circ }\right]$
0	0.96	0.64
45	1.75	1.13
90	5.65	0.96
135	3.98	1.13
180	3.07	4.74
225	2.87	0.64
270	6.31	0.66
315	7.21	13.34
**summary**	**3.72**	**4.73**
